# Targeting IRF5 inhibition in human B cells: identification of new functional roles that implicate IRF5 in systemic lupus erythematosus B-cell pathology

**DOI:** 10.1186/ar4632

**Published:** 2014-09-18

**Authors:** Saurav De, Di Feng, Alisha Valdez, Amy Pitler, Betsy J Barnes

**Affiliations:** 1 Rutgers Biomedical and Health Sciences, Newark, NJ, USA; 2 New Jersey Medical School - Cancer Center, Rutgers Biomedical and Health Sciences, Newark, NJ, USA; 3 New Jersey Medical School, Rutgers Biomedical and Health Sciences, Newark, NJ, USA

## Background

The interferon regulatory factor 5 (*IRF5*) systemic lupus erythematosus (SLE) risk loci is considered one of the most strongly and consistently associated SLE loci identified. It has been detected using both candidate gene and genome-wide association studies. Haplotypes are associated with increased, decreased, or neutral levels of risk for SLE and have been shown to associate with functional changes in IRF5-mediated signaling, including increased expression and elevated IFNα activity. The majority of studies, however, were performed in peripheral blood mononuclear cells and thus little is known of the function of IRF5 in specific human immune cell populations. We are interested in understanding the role of IRF5 in human B cells since previous studies in mice implicated a role for IRF5 in effector B-cell development and function and murine models of lupus lacking the *Irf5 *gene showed reduced ANA, glomerulonephritis and pathogenic autoantibody production. Unfortunately, many of these studies were complicated by the finding of a secondary mutation in the *Dock2 *gene amongst *Irf5^-/- ^*mice. Recent findings from our laboratory indicate that IRF5 is constitutively localized to the nucleus of human SLE memory B cells and that activation of healthy donor B cells results in IRF5 nuclear localization, suggesting a functional role for IRF5 in human B cells.

## Methods

Human immortalized and primary B cells were utilized in this study. Primary naïve B cells were obtained by informed consent at University Hospital, Newark, NJ, USA under an approved IRB protocol, and either mock stimulated or stimulated with anti-IgM and CpG-B for activation. B-cell development, cytokine expression, autoantibody production and class switch recombination were examined in the presence or absence of siRNAs targeting IRF5 expression or peptide inhibitors targeting IRF5 nuclear localization. An IRF5-mediated B-cell signature was also examined under similar experimental conditions by ChIP-seq analysis.

## Results

We find that IRF5 is indeed important for human B-cell effector functions, including, but not limited to, cytokine expression, differentiation, and autoantibody production, and have identified a pro-effector B cell gene program that is regulated by IRF5.

## Conclusions

These findings show for the first time that IRF5 is an important regulator of human effector B-cell development and function and thus, when overexpressed or overactivated, as seen in SLE B cells, would be expected to contribute significantly to SLE B-cell pathology. These data provide initial insight into how inhibitors of IRF5 activation may change SLE disease onset and/or progression.

